# Cancer Cures—Formulæ

**Published:** 1874-08

**Authors:** Thos. E. Wakefield


*MEDICAL AND SURGICAL REPORTER*—*July*, 1874:

Cancer Cures.—*By Thos. E. Wakefield, M.D.*—I give them to the profession in order that they may head off those cancer quacks who live upon the fears and credulity of mankind, everything coming beneath their notice classed as cancers, from a seed wart up to a bona fide cancer. It was purchased by one of their patients, from one of the most successful cancer doctors that ever visited this section of the country. And as far as I have known and seen its action, it is one of the most effectual and reliable of any recipe, to my knowledge, in print. I give them *verbatim et literatim:*

| | |
|---|---|
| ℞.—Arsenic, . . . . . . . . . | |
| Rochell Salts, . . . . . . . | |
| White Vitrol, . . . . . . . . | |
| Sulphur, . . . . . . . . | aa. |

Mix with yolk of eggs to the consistence of batter, and put into a new earthen dish; put into a brick or stone oven; slowly bake it until it rises up higher than the top of the dish, like a well done, rich cake; let it cool and rub up fine; mix a little of the above with the yolk of an egg and apply to the cancer. Change it every two days; do not make the plaster quite as large as the cancer, for it will find the whole of the cancer, let it extend ever so far.

Salve to use after the cancer is out:

| | |
|---|---|
| ℞.—Fresh butter or lard, . . . . | ℔ j. |
| Beeswax, . . . . . . . . | ℥ iv. |
| Pine turpentine, . . . . . . | ℥ vj. |
| Pure honey, . . . . . . . | ℥ ij. |
| Resin, . . . . . . . . . | ℥ iij. |

Melt all together, and set off the vessel from the stove, and partly cool; add half an ounce of finely pulverized verdigrease; stir until cool. This salve is to be used first, afterwards the following, to heal the sore:

| | |
|---|---|
| ℞.—Hog's lard, . . . . . . . | ℥ iv. |
| Beeswax, . . . . . . . . | ℥ ivss. |

Melt together and stir until cool.

Also for cancer (a middle recipe:)

| | |
|---|---|
| ℞.—White vitrol, . . . . . . . | 2 parts. |
| Arsenic, . . . . . . . . | 1 part. |
| Corrosive sublimate, . . . . . | ½ part. |

Mix this powder with simple cerate. Mix well and apply a little of this to the cancer every day, until it is out, and heal it with the healing salve. Also a syrup made from the following ingredients: Sassafras, guaiac wood, mandrake, yellow dock, blue flag, Turkey pea, stillingia and toad plantin.

N. B.—I don't take any stock in the syrup; it is given only to amuse the patient.

By the addition of q. s. of morphine it deprives the plaster of that excessive pain attending its use.

I hope that physicians will try the above recipes, and save their patients the trouble of going to Rome, New York, and other cancer doctors. And if their patients will have their warts, cancers, etc., (or whatever else they have,) *eat out*, let the regular profession do it.

